# Evaluation of RECIST v1.1 for predicting overall survival in sarcoma patients with pulmonary metastasis

**DOI:** 10.1186/s40644-026-01012-0

**Published:** 2026-02-26

**Authors:** Lukas Gold, Konstantin Klambauer, Christian Dascalescu, Luca Klinge, Michael Winkelmann, Nabeel Mansour, Dirk Mehrens, Jens Ricke, Michael von Bergwelt-Baildon, Dorit Di Gioia, Lars H. Lindner, Wolfgang G. Kunz

**Affiliations:** 1 Department of Radiology, University Hospital, LMU Munich, Marchioninistr. 15, 81377 Munich, Germany; 2 German Cancer Consortium (DKTK) and Bavarian Center for Cancer Research (BZKF), partner site Munich, Munich, Germany; 3 Comprehensive Cancer Center München-LMU (CCCM LMU ), LMU Munich, Munich, Germany; 4 Department of Medicine III, University Hospital, LMU Munich, Munich, Germany

**Keywords:** Sarcoma, Chemotherapy, Lung, Metastasis, RECIST

## Abstract

**Purpose:**

Response assessment in the treatment of metastatic sarcoma primarily depends on imaging, as no established clinical or serological biomarkers reliably predict survival outcomes. This study evaluates the utility of Response Evaluation Criteria in Solid Tumors (RECIST v1.1) in predicting overall survival (OS) in sarcoma patients with pulmonary metastases.

**Methods:**

We selected consecutive study subjects from a prospective registry based on the following criteria: (1) available CT imaging at first diagnosis of pulmonary metastases from sarcoma, (2) available follow-up CT imaging within 16 weeks of systemic therapy initiation, (3) documentation of OS. Volumetric segmentation of up to 5 lung metastases was performed over time. Progressive disease (PD) was defined as increase of the unidimensional sum of lesions ≥ 20% or appearance of new metastases according to RECIST v1.1. Kaplan-Meier survival analyses were performed. P values < 0.05 were considered statistically significant.

**Results:**

Ninety-two patients were included (median age: 58 years; 50% female). Average time of follow-up CT was 67 days after baseline imaging. Patients with PD on first follow-up imaging (*n* = 24; 26%) showed significantly shorter OS (13.9 months vs. 29.3 months; *p* = 0.014). The unidimensional growth threshold of 20% proposed by RECIST did not stratify OS (14.6 months vs. 26.8 months, *p* = 0.221). The appearance of new metastases (*n* = 16; 17%) indicated significantly shorter OS (7.8 months vs. 27.0 months; *p* < 0.001) and was frequently observed even in patients with decreasing size of existing metastases (*n* = 7; 8%).

**Conclusion:**

Imaging progression patterns of pulmonary metastatic sarcoma demonstrate distinct associations with OS, highlighting the need for sarcoma-specific adaptations to established response criteria.

**Supplementary Information:**

The online version contains supplementary material available at 10.1186/s40644-026-01012-0.

## Introduction

Soft tissue sarcomas with distant metastasis have a poor overall survival (OS) [1]. Common sites of metastasis vary significantly depending on the histological subtype, yet the most common sarcoma types frequently metastasize to the lung [2]. The incidence of lung metastasis varies by subtype and the imaging appearance of lung metastasis can differ among the subtypes [3]. Aside from the specific histopathological sarcoma subtype, other risk factors for developing lung metastasis that have been reported are the tumor stage and high-grade histopathology [4]. 

The presence of lung metastasis was determined to be approximately 20–25% of all patients with soft tissue sarcomas [5]. Lung metastases have been demonstrated to negatively impact patient prognosis [6], and the size of the largest lung lesion has been identified as a prognostic factor [5]. Radiologic imaging for lung surveillance has therefore become the standard of care [7]. If possible, the complete resection of lung metastases is associated with improved overall survival [8], and this approach has hence been underlined in the consensus guidelines on metastatic retroperitoneal sarcoma by the Trans-Atlantic Retroperitoneal Sarcoma Working Group (TARPSWG) [9]. 

In sarcoma patients with lung metastasis that receive systemic therapy, response assessment is currently based on radiological imaging [9], as there are no established serological markers for therapy monitoring or prognostication. The most widely applied Imaging Response Evaluation Criteria in Solid Tumors (RECIST v1.1) have originally developed based on studies in other solid malignancies and have not been specifically adapted or validated for sarcoma [10]. In contrast, modifications of RECIST have been implemented for several tumor entities to better capture their unique imaging characteristics and prognostic features, such as gastrointestinal stromal tumors, hepatocellular carcinoma, and mesothelioma. [11–13].

Evidence from the literature regarding sarcoma-specific evaluation of RECIST is limited. In osteosarcoma, for example, studies have demonstrated that RECIST-defined progressive disease in primary tumors is associated with worse overall survival (OS), but the applicability of RECIST to pulmonary metastases could not be evaluated due to insufficient patient numbers [14]. These findings highlight a critical knowledge gap and underscore the need for further evaluation of RECIST in metastatic sarcomas, particularly in the lungs, where distinct growth patterns and treatment responses may not be adequately reflected by current criteria.

The present retrospective, single-center study aims to assess the prognostic value of RECIST v1.1 parameters for OS in sarcoma patients with lung metastases and to explore whether specific imaging parameters could provide additional prognostic information for this rare and heterogeneous disease.

## Methods

### Study design and population

The study population was based on a prospective registry of all consecutive sarcoma patients who were treated at the Comprehensive Cancer Center Munich-Ludwig-Maximilian University Munich (CCCM^LMU^) between 01.01.2016 and 01.03.2022. The following inclusion criteria were applied:

1. Documentation of overall survival.
2. Available CT imaging at first diagnosis of pulmonary metastases from soft tissue or bone sarcoma.
3. Initiation of systemic therapy within 60 days after baseline CT imaging.
4. Available follow-up CT scan within 16 weeks after start of systemic therapy.

**Fig. 1:**
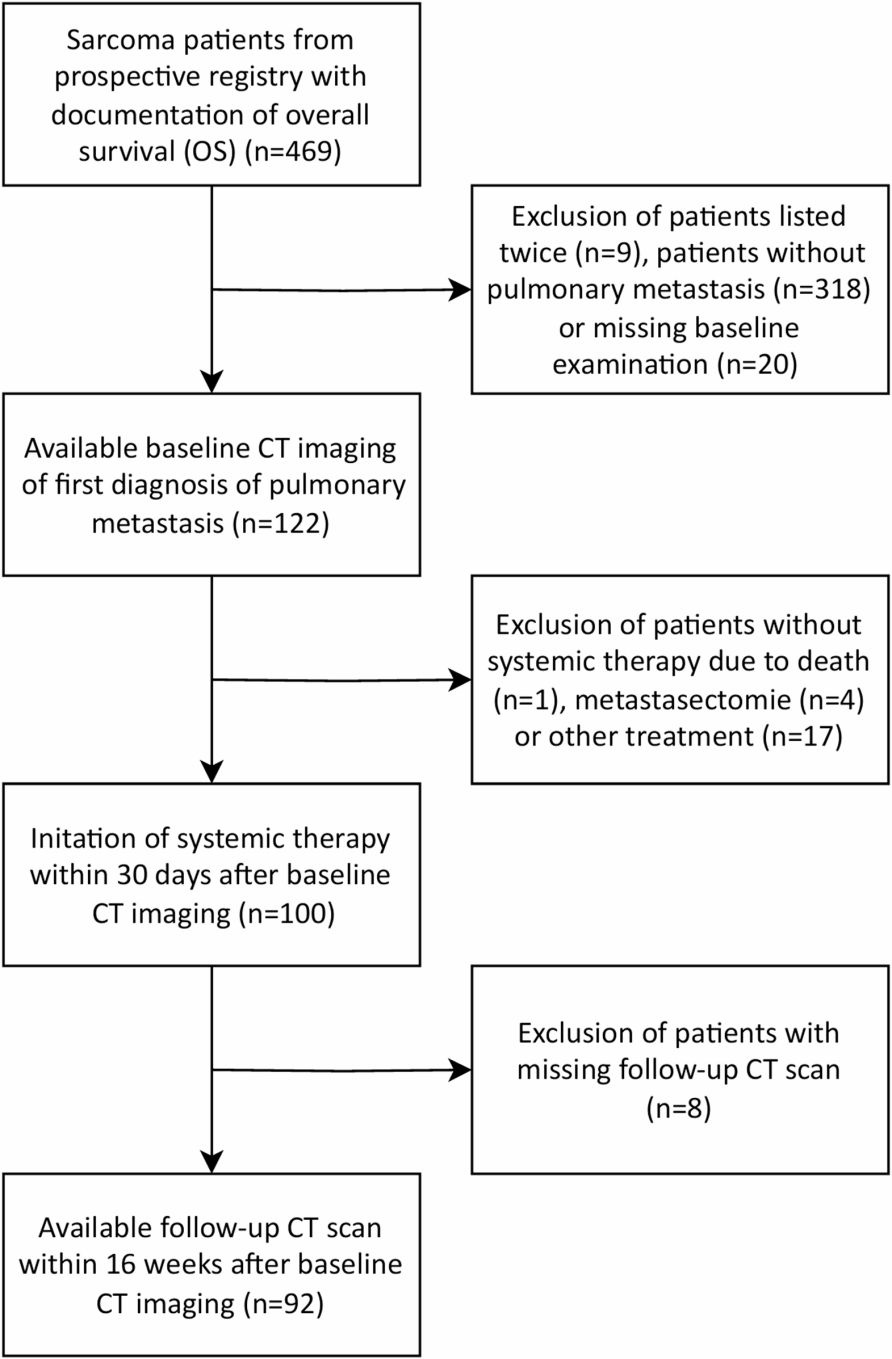
Flow diagram of patient selection

For detailed description of the selection process with reasons for exclusion see Fig. 1.

The following clinical data was extracted from the central database of the CCCM^LMU^:

1. Demographics of patients: Sex, date of birth, date of death.
2. Imaging related data: Date of baseline and follow-up CT imaging, date of imaging with tumor progression, radiology reports of baseline and follow-up CT imaging.
3. Therapy related data: Date of initiation of systemic therapy.
4. Histopathological tumor information: Grading and tumor entity.

All medical records and imaging studies were reviewed with the approval of the LMU Munich Institutional Review Board (LMU Ethics Committee, project number 22–0321). The requirement for informed consent was waived by the institutional review board due to the retrospective design and the use of data from deceased patients. All patients were treated according to the Declaration of Helsinki.

### Imaging and response assessment

Imaging analysis was performed by dedicated trial reporting software mintLesion 3.8 (mint Medical GmbH; Heidelberg, Germany). Pixel-accurate delineation was performed exclusively using the freehand tool. Metastases were manually segmented in all available CT-slices without applying the interpolation function. In patients with multiple lung metastases, a maximum of five metastases per CT scan were annotated and the metastases with the largest volume were chosen. Close attention was paid to metastases which were visually not easy to delineate standing in contact to structures as the parietal pleura, pulmonary vessels or other metastases. In some cases, metastases infiltrated the bronchia which was respected within the annotation procedure. Metastases with a volume ≤ 0.01 cm^3^ were not considered which was especially relevant for only retrospectively clearly identifiable metastases in baseline CT images after examination of the follow-up CT scans. Selection of metastases was correlated to corresponding radiology reports and further acquired imaging as previous or later performed CT scans. After agreeing on a standardized process, segmentation of baseline and follow-up CT scans was conducted in consensus reading by two persons with vast experience in medical annotation (L.K. and L.G). All segmentations were proofread by two radiologists with 3 respectively 9 years of experience (K.K. and W.G.K.). The software automatically carried out longitudinal volumetry. Response assessment was determined based on Response Evaluation Criteria in Solid Tumors version 1.1 (RECIST1.1) with progressive disease (PD) defined as increase in diameter of lesions ≥ 20% or appearance of new metastases [10]. 

### Statistical analysis

All statistical analyses were performed using open-source software (R version 4.3.1; R Foundation for Statistical Computing, Vienna, Austria). For survival analysis, OS was visualized using Kaplan-Meier survival curves. Log-rank (Mantel-Cox) test was performed to examine the significance of the results. P values below 0.05 were considered to indicate statistical significance.

## Results

### Patient characteristics

92 out of 469 patients met the inclusion criteria (median age: 58.0 years; 50% female). The three most common sarcoma subtypes were leiomyosarcoma (*n* = 18; 20%), synovial sarcoma (*n* = 12; 13%) and liposarcoma (*n* = 8; 9%). Histopathological grading was 2 for *n* = 26 (28%) patients and 3 for *n* = 37 (40%) patients. For the other patients, grading was not available (*n* = 17; 18%) or not specifically defined (*n* = 12; 13%). Detailed patient characteristics are shown in Table 1.

**Table 1:** Patient characteristics

Sex, *n* (%)	
Male Female	46 (50%) 46 (50%)
Age, years	
Median Range	58.0 18–91
Sarcoma subtype, n (%)	
Leiomyosarcoma Synovial sarcoma Liposarcoma Pleomorphic sarcoma, undifferentiated Chondrosarcoma Osteosarcoma Ewing sarcoma Angiosarcoma Solitary fibrous tumour, malignant Malignant peripheral nerve sheath tumour Rhabdomyosarcoma Myxofibrosarcoma Spindle cell sarcoma Undifferentiated sarcoma Others	18 (20%) 12 (13%) 8 (9%) 7 (8%) 6 (7%) 5 (5%) 5 (5%) 5 (5%) 4 (4%) 4 (4%) 3 (3%) 2 (2%) 2 (2%) 2 (2%) 9 (10%)
Grading, n (%)	
2 3 2/3 or “high grade” NA	26 (28%) 37 (40%) 12 (13%) 17 (18%)
Extrapulmonary metastases, n (%)	
Yes No	59 (64%) 33 (36%)
Location of extrapulmonary metastases, n (%)	
Lymph nodes Bone Liver Soft tissue Brain Other	23 (25%) 19 (21%) 19 (21%) 12 (13%) 6 (7%) 11 (12%)
Timing of lung metastasis relative to primary tumor diagnosis, n (%)	
Synchronous	49 (53%)
Metachronous	43 (47%)

### Imaging characteristics

Vast majority of scans were performed using intravenous contrast agents and reconstructed with thin slices of ≤ 3 mm. Segmentation was also considered reliable in the remaining scans with slightly thicker slices and in non-contrast CTs. The scans were predominantly acquired in-house during the portal venous phase, approximately 60 s after intravenous administration of iomeprol 350 mgI/mL (Iomeron, Bracco Imaging SpA, Milan, Italy) at a dose of 1.5 mL/kg body weight. Imaging was conducted in-house on one of three dual-source CT systems: SOMATOM Flash or SOMATOM Drive (256 slices) or SOMATOM Force (384 slices; all Siemens Healthineers AG). Tube voltage and current were automatically adjusted to patient size using CARE Dose 4D and CARE kV technologies. Collimation settings were 2 × 128 × 0.6 mm for the Flash and Drive scanners, and 2 × 192 × 0.6 mm for the Force scanner. Detailed information about technical details regarding the baseline and follow-up CT scans can be found in the Supplementary Material.

Average time of first follow-up CT was 67 days after baseline CT scan and 53 days after start of systemic therapy. The majority of patients (*n* = 46; 50%) had five or more lung metastases at baseline CT imaging, followed by two metastases (*n* = 21; 23%), four metastases (*n* = 10; 11%), one metastasis (*n* = 8; 9%) and three metastases (*n* = 7; 8%). At follow-up CT imaging this distribution changed, still most patients had (*n* = 35; 38%) five or more metastases. The number of patients with appearance of new metastases (*n* = 16; 17%) was similar to the number of patients with disappearance of existing metastases (*n* = 20; 22%) whereas most commonly the number of metastases did not change (*n* = 52, 61%). High deviation between median and mean size of the largest lung metastasis from baseline CT scans (1.15 cm^3^ versus 19.9 cm^3^) was due to two statistical outliers (1,352 cm^3^ and 119 cm^3^). First outlier significantly decreased in size (752 cm^3^) which resulted in lower mean size of the largest metastases in follow-up CT scans (13.2 cm^3^) while median size also decreased (0.7 cm^3^). The tumor volume increased for *n* = 16 (17%) patients over 73% which is equivalent to an increase in sum of unidimensional diameters of 20% as defined in the RECIST 1.1 guidelines. *N* = 16 (17%) patients experienced a change in tumor volume between 0% and 73% and *n* = 38 (41%) patients between − 66% and 0%. For *n* = 22 (24%) patients the tumor volume decreased over 66% which matches the unidimensional growth threshold of -30% according to RECIST 1.1. Table 2 summarizes the longitudinal changes in features of the pulmonary metastasis.

**Table 2:** Longitudinal changes in features of pulmonary metastasis

Time between baseline CT and follow-up CT, days	
Average Standard deviation	67 23
Time between start of systemic therapy and follow-up CT, days	
Average Standard deviation	53 22
Number of lung metastases at baseline CT, n (%)	
1 2 3 4 5 or more	8 (9%) 21 (23%) 7 (8%) 10 (11%) 46 (50%)
Size of largest lung metastasis at baseline CT, cm^3^	
Median Mean Standard deviation	1.1 19.9 140.3
Number of lung metastases at follow-up CT, n (%)	
0 1 2 3 4 5 or more	4 (4%) 12 (13%) 15 (16%) 6 (7%) 20 (22%) 35 (38%)
Size of largest lung metastasis at follow-up CT, cm^3^	
Median Average Standard deviation	0.7 13.2 86.6
Dynamic development of metastases number at follow-up CT*	
Disappearance of at least one existing metastasis Same metastases as in baseline CT Appearance of at least one new metastasis	20 (22%) 52 (61%) 16 (17%)
Dynamic development of metastases volumes at follow-up CT**	
> (+ 73%) (0%) – (+ 73%) (-66%) – (0%) < (-66%)	16 (17%) 16 (17%) 38 (41%) 22 (24%)

* Only metastases > 0,01 cm^3^ were considered

** Tumor burden according to RECIST criteria; unidimensional thresholds were adapted to volumetric changes

### Progressive disease according to RECIST

**Fig. 2:**
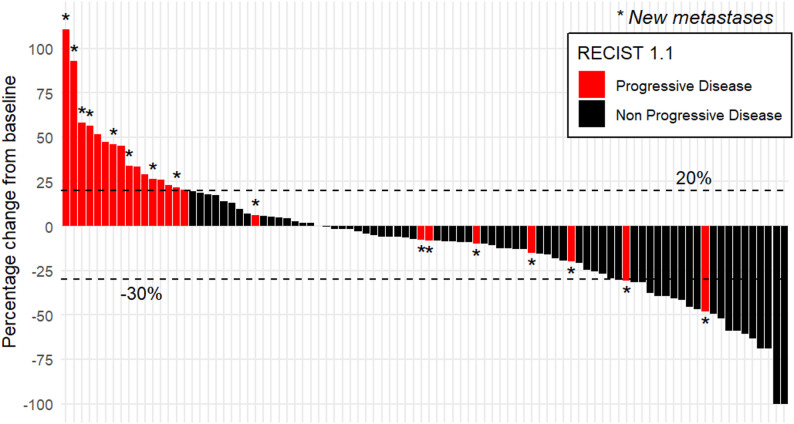
Response in growth of pulmonary metastasis under systemic therapy visualized as waterfall plot. Horizontal dotted lines mark unidimensional growth threshold for progressive disease and partial response according to RECIST 1.1. New metastases appeared in *n* = 16 patients, of which *n* = 7 patients showed a decrease in size of already existing metastases

*N* = 24 (26%) patients experienced progressive disease according to RECIST 1.1 on first follow-up imaging. The reasons for this categorization were distributed as followed: growth of existing lesions of over 20% in one dimension for *n* = 8 patients (33%) (criteria of target lesions at baseline-CT with diameter of at least 1.0 cm and subsequent unidimensional growth of over 20% were fulfilled for all *n* = 8 patients), appearance of new lesions for *n* = 8 (33%) patients, and combination of both for *n* = 8 patients (33%). Among patients with new lesions, *n* = 8 (50%) had a singular new metastasis, *n* = 1 (6%) had two new metastasis, and *n* = 7 (44%) had three or more new metastasis. The progression-free survival of the cohort was 7.05 months and the overall response rate at time of first follow-up was 30.4%. Figure 2 illustrates the changes in tumor size via waterfall plot.

### Association of tumor changes with overall survival

**Fig. 3:**
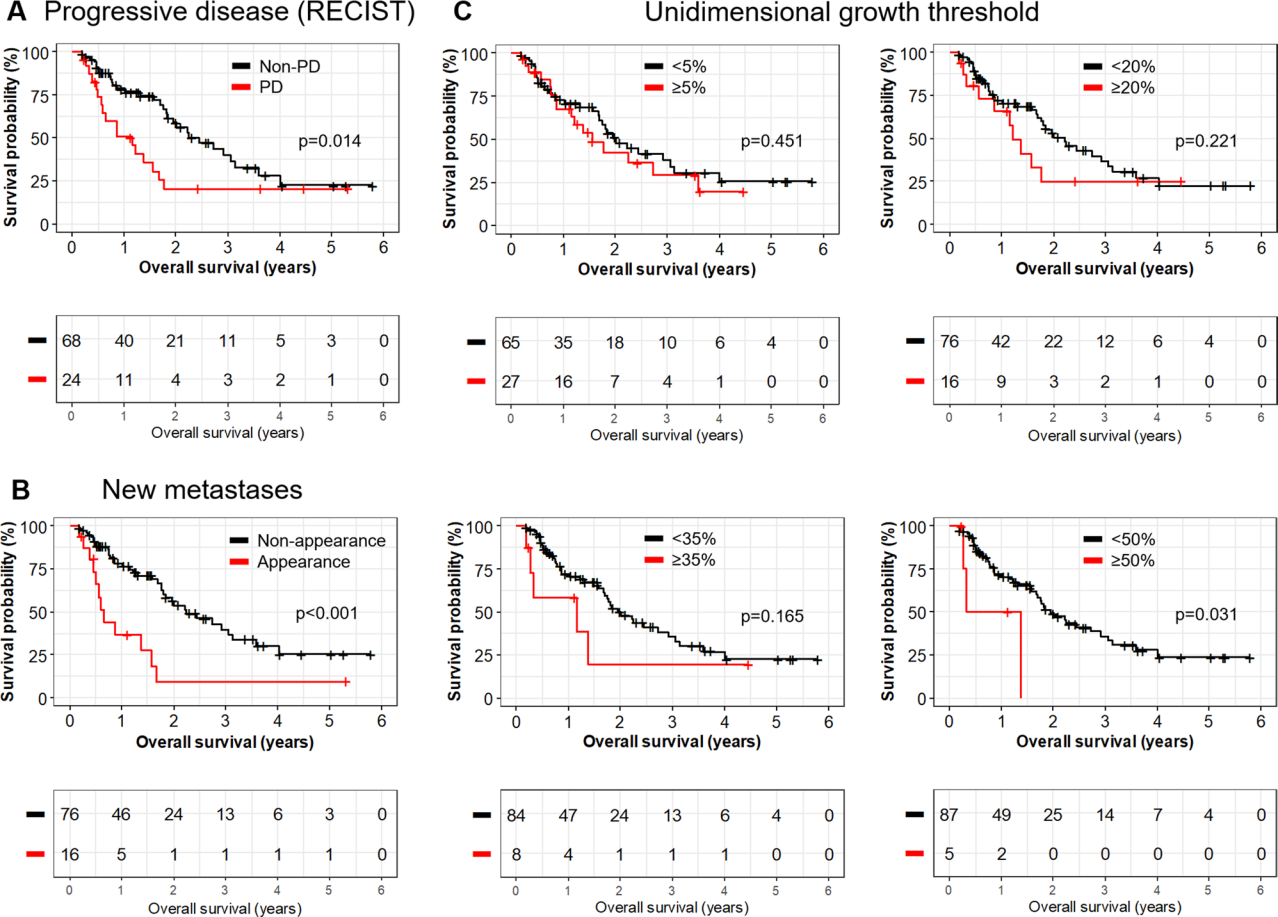
Analysis of imaged-based parameters predicting overall survival. Overall survival of patients with pulmonary metastasis for progressive disease (**A**), appearance of new metastases (**B**) and different growth thresholds (**C**)

In general, three different imaging-based items were evaluated for OS. First, patients with PD according to RECIST 1.1 showed significantly shorter OS than patients without PD (13.9 versus 29.3 months, *p* = 0.014). Second, different thresholds for unidimensional growth were tested but however were not significant for 0% (*p* = 0.155), 5% (*p* = 0.451), 10% (*p* = 0.225), 15% (*p* = 0.480), 20% (*p* = 0.221), 25% (*p* = 0.138), 30% (*p* = 0.056), 35% (*p* = 0.165), 40% (*p* = 0.165) and 45% (*p* = 0.165). Only *n* = 5 patients exceeded the growth threshold of 50% which indicated statistical significance (10.2 versus 23.5 months, *p* = 0.031). Third, development of new metastases was able to stratify OS with high statistical significance (7.8 versus 27.0 months, *p* < 0.001). Representative Kaplan-Meier survival curves are depicted in Fig. 3. Subgroup analyses for patients with histopathological osteosarcoma, soft tissue sarcoma, and for those with follow-up CT performed between 0 and 8 weeks and 9–16 weeks after initiation of systemic therapy are provided in the Supplementary Material.

### Patient examples

**Fig. 4:**
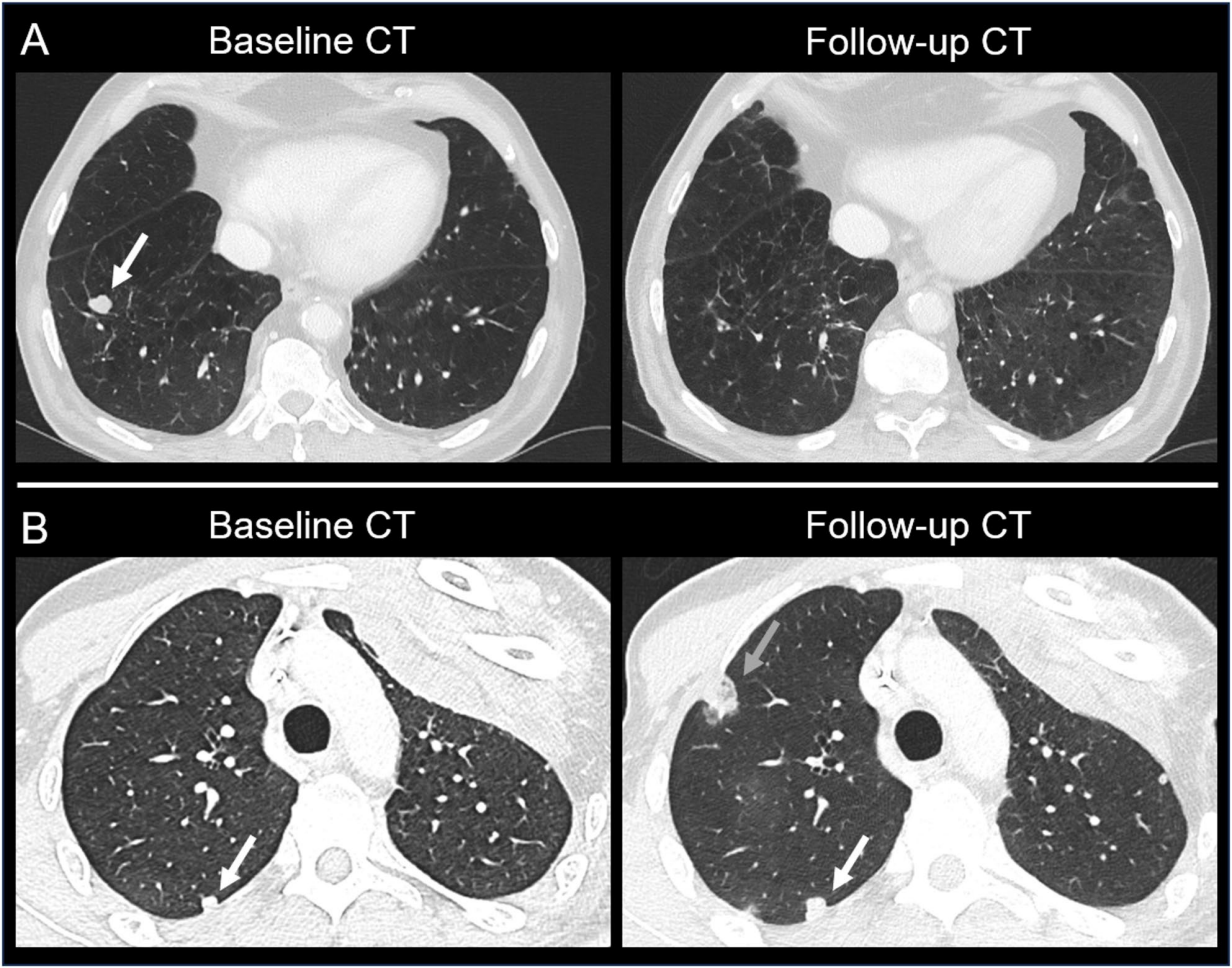
CT imaging of the lung for sarcoma patients with regressive pulmonary metastasis (**A**) and progressive pulmonary metastasis (**B**). Baseline CT of the patient shown on top revealed a singular metastasis in the right upper lobe (white arrow) which showed near-complete remission in follow-up CT after 95 days. Baseline CT of the patient shown at bottom revealed multiple metastases (*n* > 5). The white arrow points at an exemplary metastasis at the right upper lobe which had a unidimensional tumor growth of 27% in follow-up CT after 29 days. The grey arrow indicates an additional metastasis which was not present in baseline CT imaging. Overall survival was 267 (**A**) and 68 days (**B**), progression-free survival was 197 (**A**) and 29 days (**B**)

Figure 4 displays exemplary CT scans for sarcoma patients with lung metastasis. The two images on top (A) demonstrate regressive pulmonary metastasis. The singular metastasis in the right upper lobe showed near-complete remission in first follow-up CT imaging. OS of this patient was 267 days, while CT imaging revealed PD 197 days after baseline scan. On the bottom (B) an example for progressive pulmonary metastasis is shown. While baseline CT revealed multiple metastases (*n* > 5), first follow-up CT imaging indicated tumor growth of the existing metastases and appearance of new metastases. Compared to the patient above, OS and PFS were shorter in this case (197 days and 29 days)

## Discussion

In our study on patients with metastatic sarcoma to the lung, we reported the longitudinal changes in size and number of lung metastases and identified that 26% of patients had experienced progressive disease on the first follow-up timepoint based on RECIST. The absolute change in size of 20% as a RECIST threshold performed poorly as an imaging predictor for OS in this patient population. In contrast, the appearance of new lung metastasis under treatment indicated shorter OS with high statistical significance.

RECIST version 1.1 criteria have been developed by the European Organization for Research and Treatment of Cancer (EORTC) and the RECIST working group based on a large data warehouse, which included 16 trials with 6500 patients [14]. Yet, none of these trials (except one trial on GIST) have been from a clinical trial on sarcoma patients. Most solid malignancies that were included behave biologically different from soft tissue sarcomas and only few had a dominant metastatic pattern to the lung [14]. 

Since the establishment of RECIST as an imaging endpoint for clinical trials, there have been studies with several clinical trials to test the association of imaging-based parameters such as progression-free survival or overall response rate with the patients’ overall survival [15]. Aside from associations with OS in several clinical trials, these studies have also identified trials in which there was an OS benefit without improvement in imaging endpoints and even trials with worse OS despite improvement in imaging endpoints [15]. This underlines the need to test associations with OS in less represented solid malignancies, such as sarcoma patients.

In our study, the traditional growth threshold of metastasis that is used in RECIST to indicate progression of 20% performed poorly. It needs to be acknowledged that the + 20% cut-off has no biological or pathophysiological basis yet has been identified within the large data warehouse individual patient analysis of other solid malignancies. It is therefore important to study if imaging-based criteria need to be adapted for patients with metastatic sarcoma to the lung. In our cohort the growth threshold of 50% was able to stratify OS, however only *n* = 5 patients fulfilled this criterion, and this group also included *n* = 4 patients who had additional appearance of new metastases. Identifying a growth threshold that indicates a worse prognosis may be tested in clinical trials to guide therapy decisions and the optimal timepoint to switch to the next line of treatment.

In treatment monitoring of metastatic malignancies guided by RECIST, the appearance of any new lesion indicates cancer progression [10]. This dogma has biological plausibility as a newly appearing lesion clearly indicates ongoing metastasis formation and hence cancer deposits that are refractory to the ongoing systemic treatment. However, this may not always be strongly associated with poor survival as the size, location, and biology of the new lesion(s) have clinical relevance. For example, in metastatic prostate cancer, the relevance of new lesions has been studied and incorporated into modified response criteria [16]. 

In our study, the appearance of new lesions was significantly associated with OS, with patients developing new lesions showing poorer outcomes compared to those without. This finding remained consistent in subgroup analyses of patients with soft tissue sarcoma, bone sarcoma, and in those who underwent follow-up CT scans either within 0–8 weeks or 9–16 weeks after starting systemic therapy. Interestingly, while the four patients with highest tumor growth showed additional appearance of new metastases, this phenomenon was distributed rather homogeneously among the other patients of our cohort independent of metastatic growth. Nearly half of the patients (44%) with new lesions showed a decrease in size of already existing metastases. This may appear counterintuitive at first, however contemporary literature indicates that heterogeneous treatment response is common in aggressive malignancies which may also include sarcomas [17]. The biological basis may lie in the development of molecular heterogeneity within the metastatic subclones which results in diverse growth patterns of metastases under systemic therapy [18]. Site-directed biopsy of newly appearing lesions may therefore provide valuable insights into underlying molecular changes and confirm shifts in tumor biology during therapy. On a practical note, this complexity may lead to an underreporting of appearance of new metastases in patients with overall decrease in tumor burden. In our study, we used corresponding clinical reports of the CT scans only as landmarks for extraction of imaging-based parameters. Future studies should address the prognostic relevance of heterogeneous treatment response in patients with lung metastasis from sarcoma by integrating molecular and imaging-based analyses.

Our study has limitations which need to be considered. First, the relatively small sample size represents a major limitation, particularly for a study assessing survival outcomes and potential refinements to established response criteria. This concern is especially pertinent to the subgroup analyses, where smaller patient numbers further constrain statistical power. However, given that our cohort comprises patients with a rare disease, even data from a limited number of cases provide valuable insights that may inform future larger, multi-center investigations. Second, this cohort consists of different histopathological sarcoma entities which may need to be further studied to analyze if there are differential progression patterns among sarcoma subtypes. Third, the majority of patients in this cohort have also developed extrapulmonary metastases. Specifically, 59 patients (64%) presented with extrapulmonary disease involving sites such as lymph nodes, bone, liver, soft tissue or the brain. While the dominant metastatic tumor burden was primarily located in the lungs in our study cohort, the overall metastatic burden - including both pulmonary and extrapulmonary lesions - may have influenced survival outcomes. Due to the limited sample size and heterogeneity of metastatic sites, a detailed subgroup analysis comparing the impact of extrapulmonary versus pulmonary metastases on overall survival was not feasible in this study. Future investigations with larger cohorts are needed to clarify the relative prognostic significance of extrapulmonary metastatic involvement in sarcoma patients. Fourth, changes in primary tumor size under systemic therapy may be relevant predictors of OS but were not assessed in our cohort. Nearly half of the patients (47%) had metachronous lung metastases and had already undergone primary tumor resection at the time of the baseline CT. Larger and more homogeneous cohorts will be needed to investigate this question robustly.

In conclusion, our study identified items of RECIST that had different associations with overall survival. The traditional growth threshold-based approach may not be best suited in this patient cohort as we identified more prognostic value in the appearance of new metastases. A multi-center validation of our findings may warrant the adoption of response criteria for this patient population of metastatic sarcoma to the lung.

## Supplementary Information

Below is the link to the electronic supplementary material.

Supplementary Material 1

## Data Availability

The datasets generated during and/or analyzed during the current study are available from the corresponding author on reasonable request.
