# Supplementary material for: Evaluation of RECIST v1.1 for predicting overall survival in sarcoma patients with pulmonary metastasis
Source: Cancer Imaging. 2026 Feb 26;26:33. doi: 10.1186/s40644-026-01012-0 (PMC12952042; doi:10.1186/s40644-026-01012-0)
Supplement: Supplementary file 1 — Supplementary Material 1 [file 40644_2026_1012_MOESM1_ESM.pdf]

Supplementary Material

1) Technical details regarding the baseline and follow-up CT scans

	Baseline CT	Follow-up CT
In-house scan, n(%)	52 (57%)	73 (79%)
Out-of-house scan, n(%)	40 (43%)	19 (21%)
Administration of contrast agent, n(%)	90 (98%)	87 (95%)
No administration of contrast agent, n(%)	2 (2%)	5 (5%)
Exclusively CT scan, n(%)	87 (95%)	83 (90%)
Additional acquisition of positron emission tomography, n(%)	5 (5%)	9 (10%)
Slice thickness, n(%)		
1.0	13 (14%)	23 (25%)
1.5	3 (3%)	1 (1%)
2.0	27 (29%)	39 (42%)
2.5	5 (5%)	6 (7%)
3	36 (39%)	20 (22%)
4	1 (1%)	1 (1%)
5	7 (8%)	2 (2%)

Supplementary Table 1 | Technical specifications of baseline and follow-up CT imaging.

Supplementary Material

2) Subgroup survival analyses for patients with soft tissue sarcoma

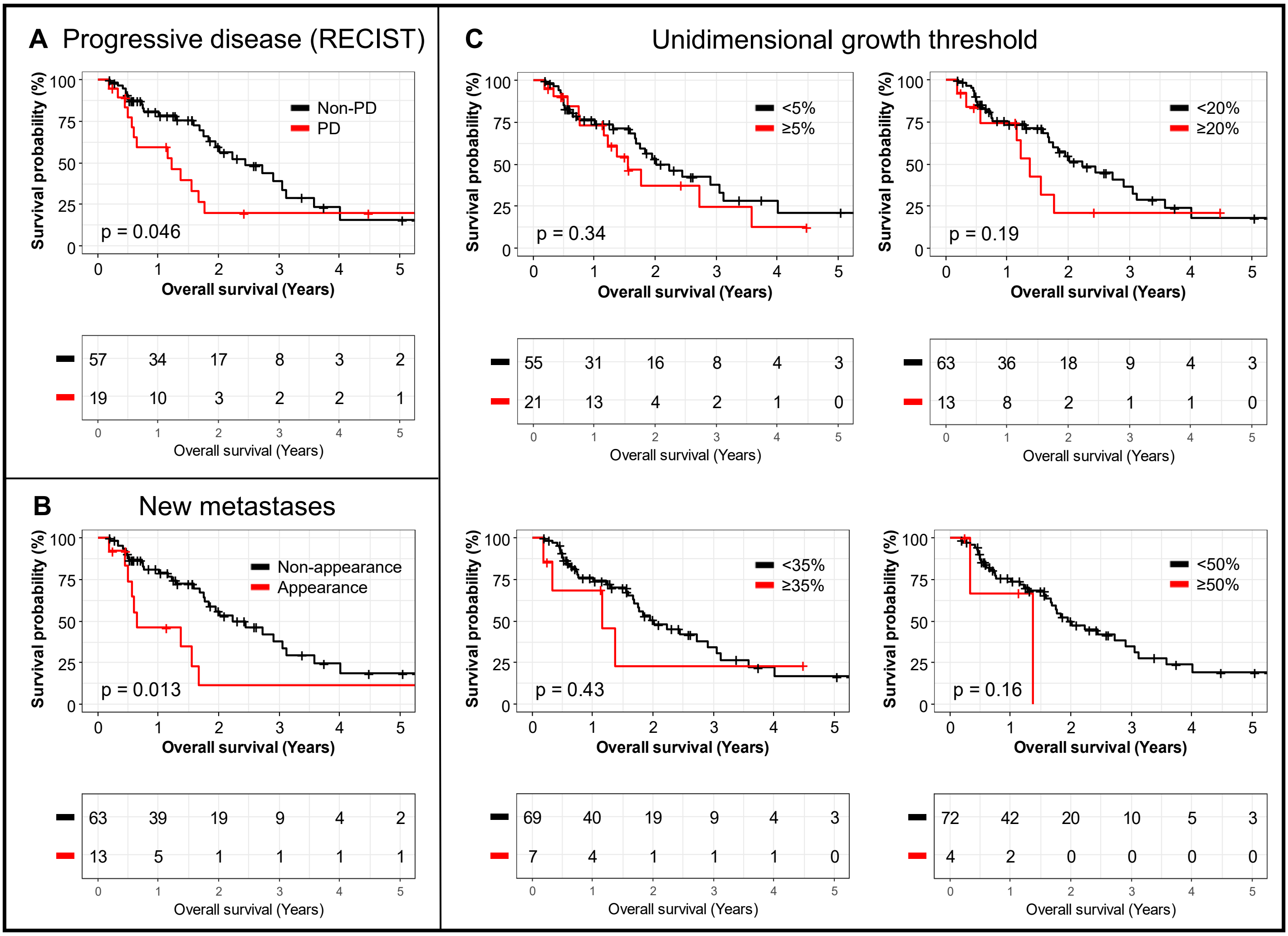

Supplementary Figure 1 | Survival analysis of imaging-based parameters predicting overall survival in patients with histopathologically confirmed soft tissue sarcoma. Kaplan-Meier curves in same arrangement as in Figure 3. RECIST-PD (A), appearance of new metastases (B) and growth thresholds of 35% respectively 50% (C) showed statistically significant results.

Supplementary Material

3) Subgroup survival analyses for patients with bone sarcoma

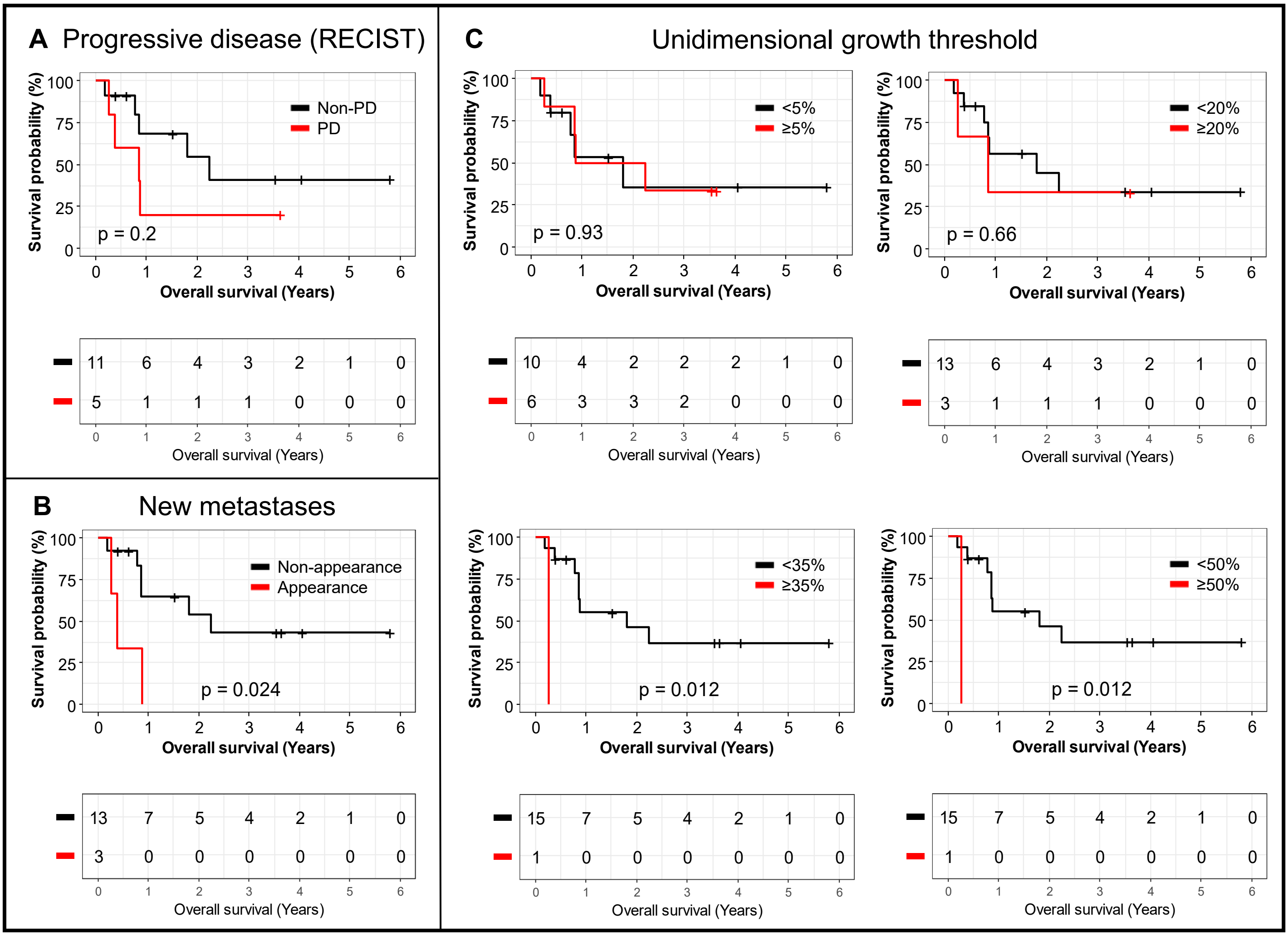

Supplementary Figure 2 | Survival analysis of imaging-based parameters predicting overall survival in patients with histopathologically confirmed bone sarcoma. Kaplan-Meier curves in same arrangement as in Figure 3. RECIST-PD (A) was not significant for OS. Appearance of new metastases (B) and growth thresholds of 35% respectively 50% (C) showed statistically significant results. Interpretability of the results is strongly limited due to small case numbers, for instance only one patient exceeded the tumor growth threshold of 35%.

Supplementary Material

4) Subgroup survival analyses for patients with a time interval of 0 - 8 weeks between start of systemic therapy and follow-up CT

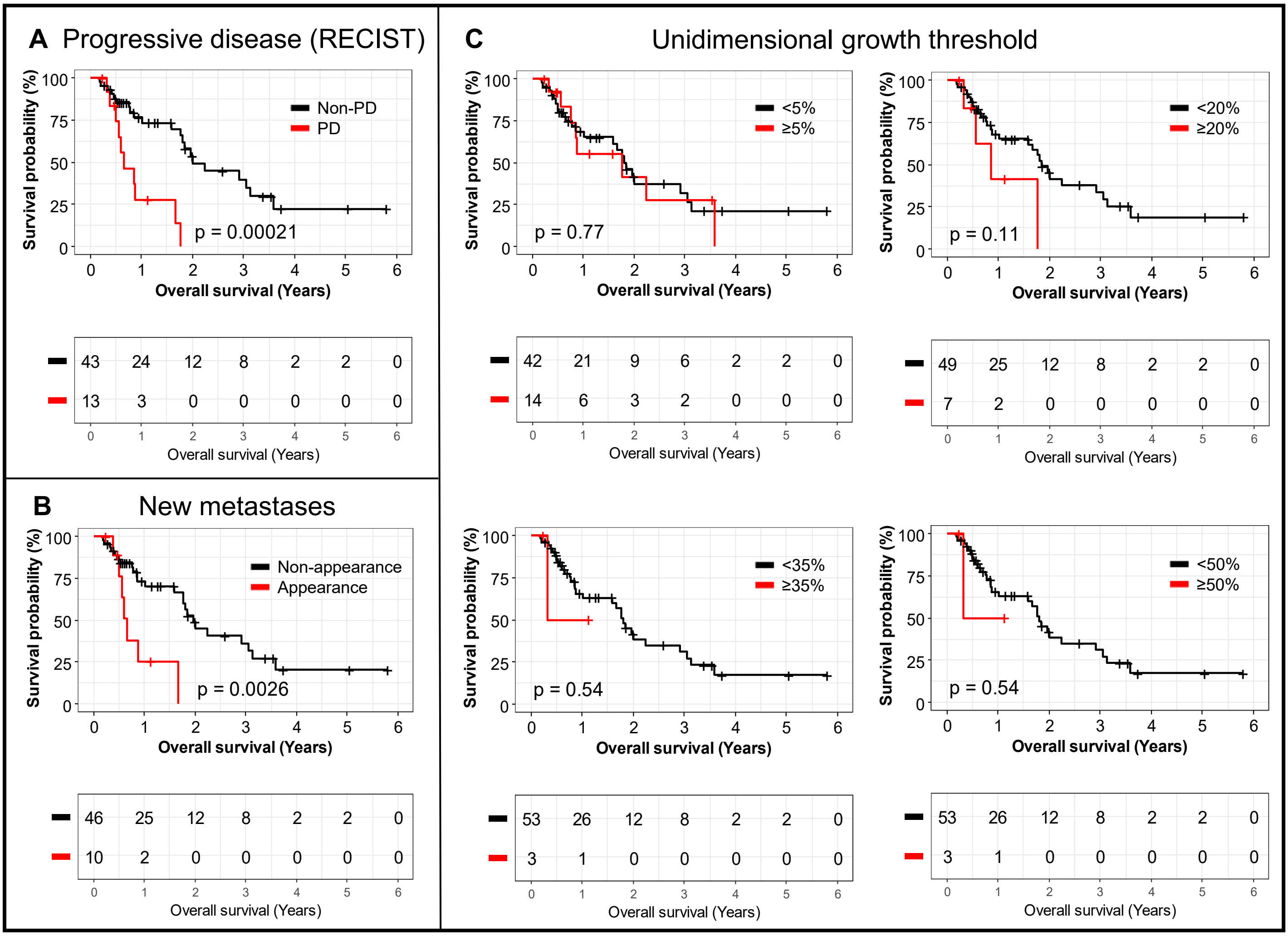

Supplementary Figure 3 | Survival analysis of imaging-based parameters predicting overall survival in patients with a time interval of 0 - 8 weeks between start of systemic therapy and follow-up CT. Kaplan-Meier curves in same arrangement as in Figure 3. RECIST-PD (A) and appearance of new metastases (B) showed statistically significant results. Tumor growth thresholds (C) did not predict OS. Interpretability of the results is limited due to small case numbers.

Supplementary Material

5) Subgroup survival analyses for patients with a time interval of 9 – 16 weeks between start of systemic therapy and follow-up CT

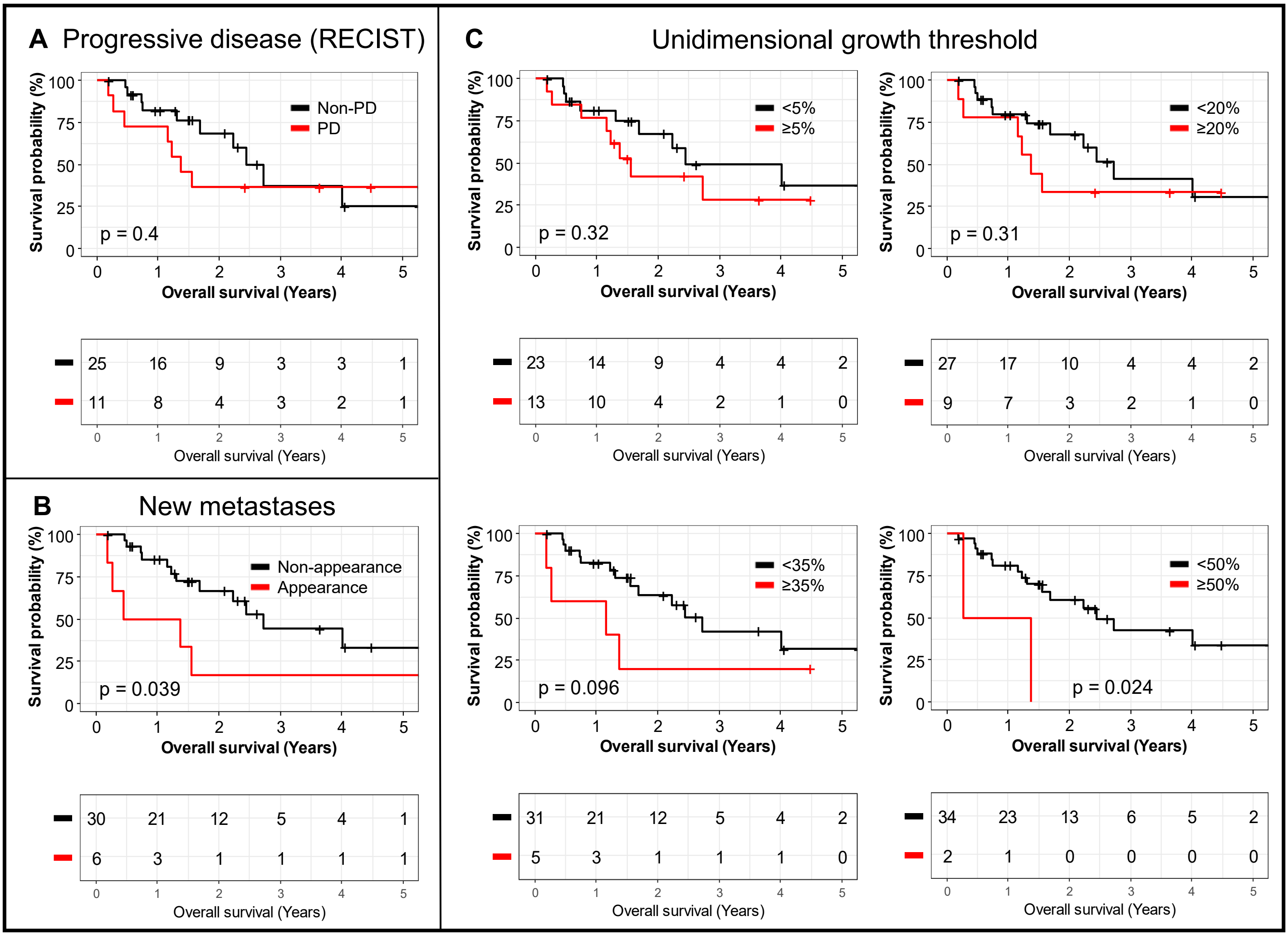

Supplementary Figure 4 | Survival analysis of imaging-based parameters predicting overall survival in patients with a time interval of 9 - 16 weeks between start of systemic therapy and follow-up CT. Kaplan-Meier curves in same arrangement as in Figure 3. RECIST-PD (A) did not predict OS. Appearance of new metastases (B) and the tumor growth threshold of 50% (C) showed statistically significant results. Classification of PD by RECIST over this relatively long interval between therapy initiation and follow-up CT may be a less accurate predictor of OS compared to classifications based on shorter time frames. However, interpretability of the results is limited due to small case numbers.
